# Effect of acupuncture for poor ovarian response: a multicenter randomized controlled trial

**DOI:** 10.3389/fendo.2026.1765527

**Published:** 2026-02-26

**Authors:** Chenchen Su, Xiaoyan Wang, Xin Liu, Li Yang, Tongsheng Su, Huidan Wang, Yu Li, Cui Zhao, Cuilian Zhang, Wenpei Xiang, Guoqing Tong, Li Chen, Fang Zhao, Huanfang Xu, Yigong Fang

**Affiliations:** 1 Institute of Acupuncture and Moxibustion, China Academy of Chinese Medical Sciences, Beijing, China; 2 Department of Acupuncture, Shaanxi Provincial Hospital of Chinese Medicine, Xi'an, Shaanxi, China; 3 State Key Laboratory of Reproductive Medicine and Offspring Health, Center for Reproductive Medicine, Institute of Women, Children and Reproductive Health, Shandong University, Jinan, Shandong, China; 4 National Research Center for Assisted Reproductive Technology and Reproductive Genetics, Shandong University, Jinan, Shandong, China; 5 Sun Yat-sen Memorial Hospital, Sun Yat-sen University, Guangzhou, Guangdong, China; 6 Reproductive Medicine Center, The University of Hong Kong-Shenzhen Hospital, Shenzhen, Guangdong, China; 7 The First Hospital of Lanzhou University, Key Laboratory for Reproductive Medicine and Embryo, Lanzhou, Gansu, China; 8 Reproductive Medicine Center, Henan Provincial People’s Hospital, Zhengzhou, Henan, China; 9 Institute of Reproductive Health, Tongji Medical College, Huazhong University of Science and Technology, Wuhan, Hubei, China; 10 Reproductive Medicine Center, Shuguang Affiliated Hospital of Shanghai University of Traditional Chinese Medicine, Shanghai, China; 11 Reproductive Medicine Center, The First Affiliated Hospital of Xi’an Jiaotong University, Xi’an, Shaanxi, China; 12 Reproductive Medicine Center, Jinling Hospital, Affiliated Hospital of Medical School, Nanjing University, Nanjing, Jiangsu, China; 13 Reproductive Medicine Department, Luoyang Maternal and Child Health Hospital, Luoyang, Henan, China

**Keywords:** acupuncture, clinical pregnancy rate, controlled ovarian hyperstimulation, poor ovarian response, randomized controlled trial

## Abstract

**Introduction:**

Acupuncture has been explored as a potential intervention for POR; however, high-quality evidence is limited. This multicenter randomized trial evaluated the effect of acupuncture on the number of oocytes retrieved following controlled ovarian hyperstimulation (COH) in women with POR.

**Methods:**

This multicenter, randomized, controlled study was conducted at nine tertiary hospitals in China between August 2018 and March 2023, with follow-up extended through March 2024. A total of 140 women aged ≤ 40 years, who met the Bologna criteria and were eligible for the antagonist ovulation induction protocol, were recruited and randomly assigned to either an acupuncture group or a control group. The acupuncture group received 36 acupuncture sessions prior to COH, while the control group received *in vitro* fertilization (IVF) only. The primary outcome was the number of oocytes retrieved. Secondary outcomes included embryological parameters, ovarian reserve markers, and clinical pregnancy and live birth rates.

**Results:**

The intention-to-treat population included 140 participants. Following intervention, the number of oocytes retrieved did not differ significantly between the acupuncture group (median [IQR]: 2.00 [1.00-3.00]) and control group (median [IQR]: 2.00 [1.00-4.00]), median between-group difference: 0.00, 95% CI [-1.00, 0.00], *p* = 0.283). Among secondary outcomes, the cleavage rate was higher in the acupuncture group than in the control group (100% vs. 87.39%; between-group difference: 12.61%; 95% CI [6.64%, 18.57%]; *p* < 0.001). Basal follicle-stimulating hormone (FSH) levels were lower in the acupuncture group compared to the control group (median [IQR]: 9.08 [6.53-12.8] vs. 11.31 [8.23-16.53]; between-group difference: -2.40; 95% CI [-4.76, -0.37]; *p* = 0.019). There were no statistically significant differences between groups in clinical pregnancy rate (34.29% vs. 21.43%; *p* = 0.090), live birth rate (21.43% vs. 15.71%; *p* = 0.385) and other prespecified outcomes. Results from the per-protocol (PP) analysis were consistent with the ITT findings. No serious adverse events were observed.

**Conclusions:**

This study did not find evidence that acupuncture significantly improves the number of oocytes retrieved in patients with POR. While it was associated with a significantly higher embryo cleavage rate and lower basal FSH levels, acupuncture did not significantly improve clinical pregnancy or live birth rates.

**Clinical Trial Registration:**

https://www.chictr.org.cn/, identifier ChiCTR1800017717.

## Introduction

Poor ovarian response (POR) is a pathological condition characterized by a diminished ovarian response to gonadotropin (Gn) stimulation during assisted reproductive technology (ART). It typically includes a reduced number of developing follicles, suppressed peak estradiol levels, an increased Gn requirement, elevated cycle cancellation rates, fewer retrieved oocytes, and lower clinical pregnancy rates (CPR) in controlled ovarian hyperstimulation (COH) cycles (1, 2). With a prevalence of 5%-35% among reproductive-aged women (3), POR leads to low cumulative pregnancy rates (10%-20% per IVF-ET cycle) (4), posing a substantial clinical challenge that adversely affects patients’ well-being and imposes considerable psychological and economic burdens.

Age is a major contributor to POR (1). Reproductive aging is characterized by a physiological decline in both the primordial follicle pool and oocyte quality, driven by hypothalamic-pituitary-ovarian (HPO) axis dysfunction and adverse changes in the ovarian microenvironment, including oxidative stress, mitochondrial dysfunction, and aberrant telomere dynamics (5, 6). In addition to this age-related decline, a pathological form of diminished ovarian reserve (DOR) can occur in some women before the age of 40 due to genetic (7), environmental (8, 9), or iatrogenic factors (10, 11), which is similarly characterized by premature follicle exhaustion and compromised oocyte quality. In both contexts, the resultant follicular dysfunction leads to the downregulation of follicle-stimulating hormone receptor (FSHR) expression in granulosa cells (12). The subsequent reduction in ovarian sensitivity to Gn and the smaller recruitable follicle cohort during COH cycles lead to elevated oocyte aneuploidy and impaired embryonic development (13), ultimately explaining the poor ART outcomes in POR (14, 15).

Current medical strategies for improving the number of oocytes retrieved in POR patients include pretreatment with growth hormone, testosterone, coenzyme Q10, or platelet-rich plasma (PRP) (16–18). Nonetheless, the 2020 ESHRE guidelines do not recommend routine use of GH or similar agents due to a lack of robust evidence (19). While PRP has shown promise in improving oocyte maturation and ovarian function (20), current studies are associated with a high risk of bias and inadequate safety data (21). Therefore, POR management remains a persistent challenge.

Acupuncture, grounded in traditional Chinese medicine (TCM) theory, has been used for millennia in treating gynecological conditions and has been explored as an adjunctive therapy in ART. Potential mechanisms by which acupuncture may benefit POR patients include regulating the HPO axis (21, 22), reducing inflammation (23), modulating miRNA expression (24), and influencing DNA methylation (25–27). Nevertheless, current clinical evidence is constrained by small sample sizes, limited numbers of studies, predominantly single-center design, and a lack of standardized treatment protocols (28–31). Therefore, we conducted a rigorous, multicenter randomized controlled trial to evaluate the effect of acupuncture on the number of oocytes retrieved and pregnancy outcomes in women with POR.

## Materials and methods

### Study design and randomization

This multicenter, randomized, controlled trial was conducted at nine tertiary hospitals in China, including Shanxi Provincial Hospital of Traditional Chinese Medicine; Reproductive Medicine Center Affiliated to the Institute of Women, Children and Reproductive Health of Shandong University; Sun Yat-sen Memorial Hospital of Sun Yat-sen University; the First Hospital Affiliated to Lanzhou University; Henan Provincial People’s Hospital; Tongji Medical College of Huazhong University of Science and Technology; Shuguang Hospital Affiliated to Shanghai University of Traditional Chinese Medicine; the Affiliated Jinling Hospital of Nanjing University; and Luoyang Maternal and Child Health Hospital. Eligible participants were randomly assigned in a 1:1 ratio to the acupuncture group or control group using a central, computer-generated randomization system (PROC PLAN, SAS version 9.4). Allocation was concealed through secure, independent storage of the scheme, and implemented at each site by a coordinator not involved in treatment or assessment. This coordinator had access to the central randomization system to retrieve the group assignment results for eligible participants. After baseline assessments and written informed consent were completed, the coordinator disclosed the allocation information to the acupuncturist responsible for treatment. Participant blinding was not possible due to the use of a no-treatment control. However, to minimize bias, all participants received a uniform study description during the consent process. Additionally, all study procedures, equipment, and diagnostic assessments were standardized and administered identically to both groups. Outcome assessors and data analysts remained blinded to the treatment allocation throughout the trial until the analysis was finalized. The study protocol was approved by the Ethics Committees (ECs) of each participating site. The trial protocol has been previously published (32). This study was conducted in accordance with the ethical principles of the Declaration of Helsinki. Written informed consent was obtained from all participants prior to enrollment.

### Participants

Patient recruitment for this study was conducted from August 2018 to March 2023, and follow-up was concluded in March 2024. A total of 341 participants were screened, among whom 140 patients were randomly assigned to the acupuncture group (n = 70) and the control group (n = 70).

The acupuncture and control groups had identical inclusion criteria, as follows: (1) age ≤ 40 years; (2) meeting the Bologna criteria for POR (33), defined as at least one previous IVF cycle with ≤ 3 oocytes retrieved following conventional stimulation, and an abnormal ovarian reserve test (AFC < 5–7 follicles or AMH < 0.5–1.1 ng/ml); (3) scheduled for a GnRH antagonist protocol; and (4) provision of written informed consent.

The exclusion criteria were as follows: (1) a history of ≥ 2 recurrent spontaneous abortions (excluding biochemical pregnancies); (2) recurrent implantation failure (failure to achieve clinical pregnancy after ≥ 3 transfer cycles with ≥ 4 high-quality transferred embryos); (3) uterine abnormalities (e.g., unicornuate, bicornuate, didelphic, or untreated septate uterus) or other conditions compromising endometrial receptivity or distorting the uterine cavity (e.g., untreated adenomyosis, submucosal fibroids, or intrauterine adhesions); (4) an abnormal karyotype in either partner (excluding chromosomal polymorphisms); (5) untreated hydrosalpinx; (6) any contraindications to ART or pregnancy, including uncontrolled medical disorders that could adversely affect pregnancy outcomes (e.g., hypertension, diabetes, hepatic or renal dysfunction, severe anemia, or a history of thromboembolism or malignancy); (7) a history of iatrogenic DOR, such as prior ovarian surgery, pelvic radiation, chemotherapy, or treatment with gonadotoxic drugs.

### Intervention

#### Acupuncture pretreatment intervention

**Figure 1:**
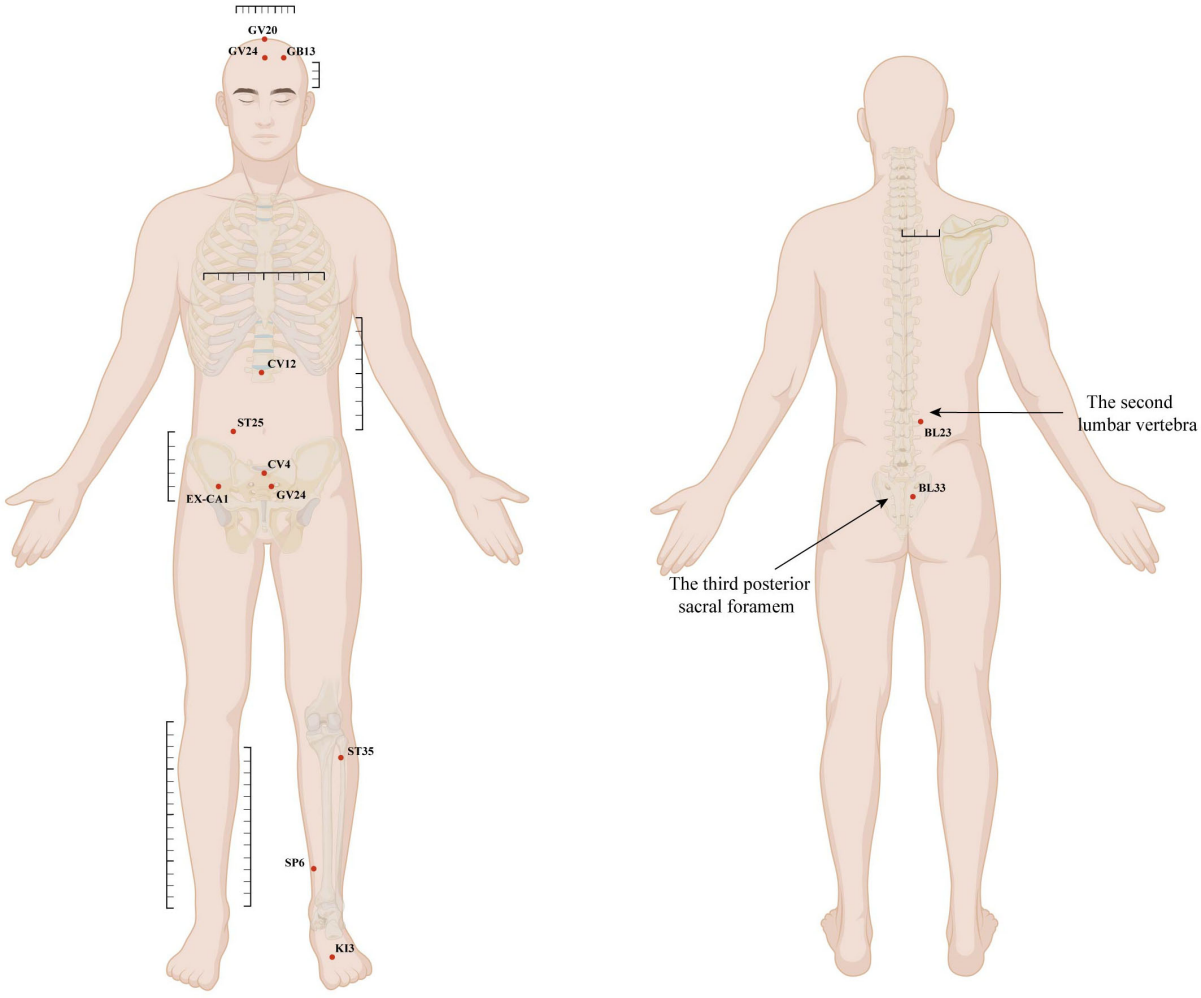
Standardized locations of acupoints used in this study.

The acupuncture group received a standardized 12-week intervention comprising three sessions per week (36 sessions total). All treatments were delivered by licensed acupuncturists (≥2 years of clinical experience) who underwent pre-trial protocol training and were not involved in recruitment, outcome assessment, or data analysis. Sterile, single-use Hwato stainless-steel needles (sizes: 0.25 × 25 mm, 0.25 × 40 mm, and 0.30 × 75 mm) were used, alongside SDZ-III electroacupuncture devices (Hwato Suzhou Medical Appliance Factory, Suzhou, China). The acupuncture protocol was divided into two sets of acupoints (**Figure 1**) based on the needling position (supine and prone). One set was used per session for 20 minutes, with the two sets alternating.

Acupuncture points were located and needled primarily in accordance with the World Health Organization Standardized Acupuncture Point Locations (34). During treatment, the sacral point BL33 was deeply needled to a depth of 60–70 mm, with the needle tip ideally advanced into the third posterior sacral foramen. Conventional needling techniques were applied to all other points: head points (GV20, GV24, GB13) were subcutaneously needled to a depth of 10–20 mm; abdominal and lower-limb points (CV12, ST25, CV4, KI12, EX-CA1, ST36, SP6, KI3) were perpendicularly needled to a depth of 30–40 mm; and LR3 on the foot was perpendicularly needled to a depth of 10–20 mm. Following insertion, all needles were uniformly manipulated using lifting, thrusting, or twirling techniques to elicit deqi, a composite needle sensation (soreness, numbness, distention, or heaviness) considered essential for achieving therapeutic effects. For treatments in the prone position, paired electrodes from an electroacupuncture device were connected transversely to the needle handles at bilateral BL23 and BL33, using a dense-disperse wave mode at a tolerable intensity.

#### Non-treatment control

The control group received no acupuncture pretreatment before COH.

#### IVF-ET procedure

Both groups of participants commenced the GnRH antagonist protocol 2–3 days into the subsequent menstrual cycle. On menstrual day 2 or 3, Gn administration (75–300 IU) was initiated after confirming baseline ovarian status via ultrasound and serum hormone level assessments. After 5 days of stimulation, the Gn dosage was adjusted based on ovarian response, monitored by ultrasound and estradiol levels; human menopausal gonadotropin (hMG, Menopur) was supplemented when necessary. Subsequent monitoring via ultrasound and hormone assays was conducted every 1–2 days to guide further adjustments to the Gn dose. When the leading follicles reached ≥ 12 mm in mean diameter, cetrorelix (Orgalutran, 0.25 mg/day) was administered daily until and including the day of the trigger injection. Final oocyte maturation was triggered with 4000-10,000 IU of urinary hCG or a recombinant hCG equivalent when at least three follicles measured ≥ 18 mm. Oocyte retrieval was performed 36 hours later. Fertilization was accomplished 4–6 hours after retrieval using conventional IVF or Intracytoplasmic sperm injection (ICSI). On day 3, embryos were graded according to each center’s standard criteria. The two highest-quality embryos were selected for the initial fresh or frozen transfer, and the remaining viable embryos were cryopreserved.

#### Protocol-allowed deviations

Participants were allowed to discontinue their assigned protocol early to begin a COH cycle once eligible, provided they had completed at least 4 weeks of the study. This constituted a minimum of 12 acupuncture sessions for the treatment group or 4 weeks in the no-treatment control group.

### Outcomes

#### Primary outcome

The primary outcome was defined as the number of oocytes retrieved per ovarian stimulation cycle.

#### Secondary outcomes

The secondary outcomes included: other IVF-related outcomes (fertilization rate, cleavage rate, usable embryo rate, high-quality embryo rate, and embryo implantation rate, clinical pregnancy rate [CPR], live birth rate [LBR], and miscarriage rate); ovarian reserve outcomes (antral follicle count [AFC] and levels of anti-Müllerian hormone [AMH], basal follicle-stimulating hormone [FSH], basal luteinizing hormone [LH], and basal estradiol [E_2_]); regular menstruation rate and the self-rating anxiety scale (SAS) scores. Clinical pregnancy was confirmed by ultrasound visualization of an intrauterine gestational sac following embryo transfer; c Live birth was defined as the delivery of one or more live infants at or after 24 weeks of gestation; Miscarriage was defined as spontaneous pregnancy loss before 24 weeks of gestation; All participants were followed for live birth outcomes up to 12 months after embryo transfer. Detailed definitions and calculation methods of the IVF-related outcomes are presented in **Supplementary Table S1**.

### Sample size

Sample size calculation was based on previously reported data (35) of retrieved oocytes (acupuncture: 4.88 ± 1.84 vs. control: 3.95 ± 1.66). With a two-sided α of 0.05 and 80% power, 57 women per group were needed. To account for a 20% dropout rate, the total sample size was set at 140, with 70 participants in each group.

### Statistical analysis

Statistical analyses were conducted with R software (version 4.4.1). The primary analysis was an intention-to-treat (ITT) analysis, adhering to the principle that all randomly allocated participants were encompassed in the groups to which they were assigned. Continuous variables with a normal distribution are presented as the mean ± standard deviation. Non-normally distributed continuous variables are summarized as the median and interquartile range (IQR). Categorical variables are reported as numbers and percentages. Continuous variables that were normally distributed were evaluated using independent samples t-tests, and between-group differences were summarized as mean differences alongside 95% confidence intervals (CI). Continuous variables with a non-normal distribution were contrasted via the Mann-Whitney U test, with between-group disparities quantified using the Hodges-Lehmann estimator (median difference) and 95% CI. Categorical variables were compared using Chi-square or Fisher’s exact tests, as applicable. Differences between groups are presented as absolute risk differences, accompanied by 95% confidence intervals. Sensitivity analysis underwent implementation using the Per-Protocol (PP) population. All tests were two-sided, and a *p*-value < 0.05 was considered statistically significant.

## Results

### Characteristics of the participant

**Figure 2:**
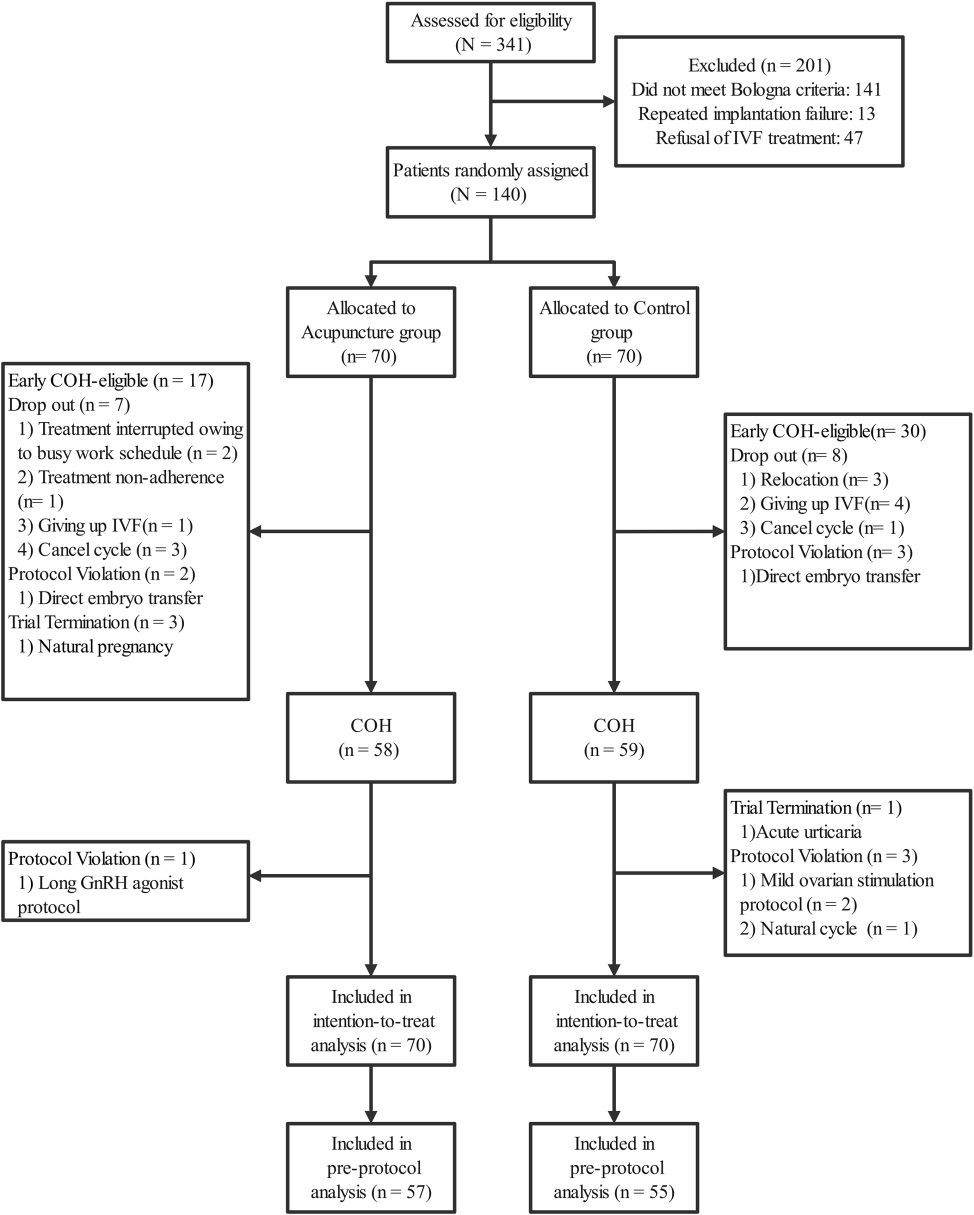
Trial flow diagram.

The participant flow diagram is presented in **Figure 2**. Among the randomized participants, 112 (80%) completed the study (57 in the acupuncture group and 55 in the control group). 17 participants in the acupuncture group and 30 in the control group initiated COH early after completing the minimum 4-week period (acupuncture treatment for the acupuncture group and a waiting period for the control group). Three participants in the acupuncture group conceived spontaneously after completing the prescribed acupuncture treatment but prior to COH initiation, and all of these pregnancies resulted in live births. No significant differences were observed between the groups in baseline characteristics, including age, reproductive history, ovarian reserve (AFC, AMH, FSH, LH, E_2_), and SAS scores (**Table 1**).

**Table 1:** Baseline characteristics of the ITT population.

Characteristics	Acupuncture group (*n* = 70)	Control group (*n* = 70)	*P Value*
Age[years, mean (SD)]	33.86 (3.52)	33.26 (3.69)	0.994
BMI[kg/m^2^, mean (SD)]^a^	23.17 (3.32)	25.70 (3.75)	0.370
Age at menarche[years, mean (SD)]	13.71 (2.54)	13.45 (1.08)	0.428
Regular menstruation [No. (%)]	21 (30)	31 (44.29)	0.080
Previous live birth history[No. (%)]	33 (47.14)	37 (52.86)	0.499
Number of oocytes retrieved (median [IQR])	2.00(1.00-3.00)	2.00(1.00-3.00)	0.707
AFC(median [IQR])	4.00(2.00-6.00)	4.00(3.00-6.00)	0.225
AMH(ng/ml, median [IQR])	0.55 (0.32-0.88)	0.62 (0.34-0.96)	0.744
FSH(IU/L, median [IQR])	9.86 (7.81-13.68)	10.30 (7.82-15.31)	0.562
LH(IU/L, median [IQR])	3.66 (2.59-4.82)	3.60 (2.47-5.66)	0.649
E_2_(pg/ml, median [IQR])	48.00 (36-76.04)	44.72 (29.92-72.07)	0.416
SAS[mean (SD)]	33.73(6.58)	33.94(6.38)	0.849

SD, standard deviation; IQR, Interquartile Range; AFC, antral follicle count; AMH, anti-Müllerian hormone; FSH, follicle-stimulating hormone; LH, luteinizing hormone; SAS, Self-Rating Anxiety Scale. ^a^BMI was calculated as weight (kg) divided by height squared (m²).

### Outcomes

#### Primary outcome

Following COH, the median number of oocytes retrieved was comparable between the acupuncture group (2.00 [1.00-3.00]) and the control group (2.00 [1.00-4.00]), with a between-group difference of 0 (95% CI[-1.00, 0], *p* = 0.283) (**Table 2**).

**Table 2:** Primary and secondary outcomes of the ITT population.

Outcome Indicators	Acupuncture group (*n* = 70)	Control group (*n* = 70)	Between-group difference (95% CI)	*P Value*
Number of oocytes retrieved[median (IQR)]	2.00 (1.00-3.00)	2.00 (1.00-4.00)	0.00(-1.00, 0.00)	0.283
Fertilization rate[No. (%)]	83.19 (94/113)	87.50 (119/136)	-4.31(-13.17, 4.54)	0.335
Embryo cleavage rate[No. (%)]	94/94 (100)	104/119(87.39)	12.61 (6.64, 18.57)	<0.001
Utilizable embryo rate[No. (%)]	77/94 (81.91)	86/104(82.69)	-0.78 (-11.43, 9.87)	1.000
High-quality embryo rate [No. (%)]	42/94 (44.68)	48/104 (46.15)	-1.47 (-15.36, 12.41)	0.948
Embryo implantation rate[No. (%)]	21/51 (41.18)	19/51 (37.25)	3.93 (-15.01, 22.86)	0.685
Total CPR[No. (%)]^a^	24/70 (34.29)	15/70 (21.43)	12.86[-1.84, 27.56]	0.090
CPR per fresh embryo transfer [No. (%)]	9/17 (52.94)	7/12 (58.33)	-5.39 (-42.01, 31.23)	0.774
CPR per FET transfer [No. (%)]	12/18 (66.67)	8/20 (40.00)	26.67 (-3.92, 57.25)	0.100
Total LBR[No. (%)]^a^	15/70 (21.43)	11/70 (15.71)	5.72[-7.13, 18.56]	0.385
LBR per fresh embryo transfer [No. (%)]	6/17 (35.29)	6/12 (50.00)	-14.71 (-50.99, 21.58)	0.423
LBR per FET transfer [No. (%)]	6/18 (33.33)	5/20 (25.00)	8.33 (-20.55, 37.22)	0.572
miscarriage rate [No. (%)]	9/21 (42.86)	4/15 (26.67)	16.19 (-14.61, 46.99)	0.484
Regular menstruation [No. (%)]	49/70 (70.00)	41/70 (58.57)	11.43(-4.33,27.19)	0.158
AFC(median [IQR])	4.00 (3.00-6.00)	4.00 (3.00-6.00)	0.00 (-1.00, 1.00)	0.937
AMH(ng/ml,median [IQR])	0.68 (0.28-1.08)	0.67 (0.3-1.15)	0.01 (-0.27, 0.26)	0.938
Basal FSH (IU/L,median [IQR])	9.08 (6.53-12.8)	11.31 (8.23-16.53)	-2.40 (-4.76, -0.37)	0.019
Basal LH (IU/L,median [IQR])	3.66 (2.59-4.82)	3.60 (2.47-5.66)	-0.16 (-0.94, 0.53)	0.649
Basal E_2_ (pg/ml,median [IQR])	57.79 (39.85-84.5)	53.00 (36.01-71.64)	6.00 (-5.10, 20.00)	0.308
SAS [mean(SD)]	31.23 (6.43)	32.62 (7.50)	-1.39 (-3.85, 1.07)	0.234

SD, standard deviation; IQR, Interquartile Range; CPR, clinical pregnancy rate; LBR, live birth rate; AFC, antral follicle count; AMH, anti-Müllerian hormone; FSH, follicle-stimulating hormone; LH, luteinizing hormone; SAS, Self-Rating Anxiety Scale; ^a^ The three cases of natural clinical pregnancy in the acupuncture group were included in the calculation of total CPR and total LBR.

#### Secondary outcomes

The cleavage rate was significantly higher in the acupuncture group than in the control group (100% vs. 87.39%, between-group difference: 12.61%, 95% CI [6.64%, 18.57%], *p* < 0.001). No statistically significant differences were observed between the acupuncture and control groups in total clinical pregnancy rate (34.29% vs. 21.43%; difference: 12.86%, 95% CI: -1.84 to 27.56; *p* = 0.090), total live birth rate (21.43% vs. 15.71%; difference: 5.72%, 95% CI: -7.13 to 18.56; *p* = 0.385). The remaining IVF outcomes (embryo implantation rate, fertilization rate, usable embryo rate, high-quality embryo rate, CPR per fresh embryo transfer, clinical pregnancy rate per FET, LBR per fresh embryo transfer, live birth rate per FET, and miscarriage rate) showed no significant differences between the two groups (**Table 2**).

Following the intervention, basal FSH levels were significantly lower in the acupuncture group compared to the control group (median [IQR]: 9.08 [6.53-12.8] vs. 11.31 [8.23-16.53], between-group difference: -2.40, 95% CI [-4.76, -0.37], *p* = 0.019). No significant intergroup differences were observed in ovarian reserve outcomes (AFC, AMH, LH, E_2_); regular menstruation rate and SAS scores (**Table 2**). Results of the per-protocol (PP) analysis aligned with those of the ITT analysis (**Supplementary Table S2**).

Notably, three participants in the acupuncture group achieved natural pregnancy after treatment, and all of these pregnancies resulted in live births, an outcome not observed in the control group (**Supplementary Table S3**).

### Adverse events

The AEs are summarized in **Table 3**. Throughout the study, no serious AEs were reported, with only 6 non-serious AEs recorded. In the acupuncture group, 5 participants reported 5 AEs, including 4 acupuncture-related AEs (4 cases of subcutaneous hematoma) and 1 intervention-unrelated AE (herpes zoster). In the control group, one participant reported 1 intervention-unrelated adverse event (acute urticaria). All intervention-related AEs were both mild and transient.

**Table 3:** AEs related and unrelated to treatment.

Adverse Event		Acupuncture group (n=70)	Control group (n=70)
Overall[No. (%)]		5 (7.14)	1 (1.43)
Serious adverse event[No. (%)]		0	0
Related to treatment[No. (%)]	Subcutaneous hematoma	4 (5.71)	0
Unrelated to treatment[No. (%)]	Herpes zoster	1 (1.43)	0
	acute urticaria	0	1 (1.43)

## Discussion

This multicenter, randomized, controlled trial involving 140 patients with POR did not demonstrate a significant difference in the number of oocytes retrieved between the acupuncture and control groups. No significant differences were found in most other secondary outcomes, including some ovarian reserve markers (AMH, AFC, LH, E_2_), some IVF parameters (fertilization rate, usable embryo rate, High-quality embryo rate, Embryo implantation rate, CPR, LBR) and anxiety scores. However, acupuncture statistically significantly improved the embryo cleavage rate and reduced the basal levels of FSH.

Our investigation adds to the ongoing debate regarding acupuncture’s efficacy for POR. This study could not confirm a significant benefit of acupuncture on the number of oocytes retrieved in POR patients. This finding positions our results within the context of conflicting evidence in the field, aligning with null results (26, 36) but contradicting the positive outcomes reported in a meta-analysis (28) and individual RCTs (30, 37). Two major factors may explain this heterogeneity. Firstly, patient characteristics differed markedly: our cohort was characterized by more severe ovarian impairment: over half had AMH <0.5 ng/mL, most had AFC <5, and the median oocyte yield was only 3. This overall low ovarian reserve may have reduced the responsiveness to any intervention, potentially contributing to our inability to detect a significant treatment effect. Secondly, and perhaps more critically, acupuncture dosage may have been suboptimal in a considerable proportion of our cohort. A systematic review has highlighted that the duration and dosage of acupuncture are pivotal for its success in IVF (38). In our trial, 17 patients discontinued acupuncture after a minimum of 4 weeks to initiate COH, meaning the total number of sessions they received likely fell below the potential therapeutic threshold for influencing oocyte yield. Therefore, future investigations should expand the sample size and establish the minimal and optimal acupuncture regimen necessary to improve reproductive outcomes in POR patients. In addition, well-designed subgroup analyses based on key baseline characteristics such as age, ovarian reserve parameters, and previous treatment history may help identify specific patient subgroups that are more likely to benefit from acupuncture. Nevertheless, although the number of oocytes retrieved did not show a statistically significant improvement in this study, its multicenter design, standardized intervention protocol, and comprehensive outcome evaluation system provide a valuable and replicable methodological reference for future research in this field.

A noteworthy finding of our study was the significant improvement in embryo cleavage rate following acupuncture. In patients with POR, oocyte quality is often impaired by factors such as mitochondrial dysfunction, elevated oxidative stress, and increased inflammatory levels, which in turn impairs oocyte chromosomal integrity and embryonic developmental potential (39, 40). Notably, cleavage is the earliest stage of embryonic development, defined as the process by which the zygote undergoes rapid mitotic divisions to produce smaller cells termed blastomeres. The cleavage rate directly reflects the mitotic activity and metabolic function of the zygote, serving as a key indicator for assessing embryonic viability, morphological quality, and blastocyst formation potential (41–43). Therefore, enhancing the cleavage rate consequently expands the pool of viable embryos with developmental potential available for selection during early development, which in turn may improve subsequent embryonic development outcomes.

Previous studies have shown that acupuncture can improve the overall embryonic quality in patients with POR, specifically manifested by increased usable embryo rate and high-quality embryo rate (27, 44). Although this study did not observe a significant improvement in the usable embryo rate or high-quality embryo rate following acupuncture intervention, the elevated cleavage rate provides key evidence that acupuncture can enhance early embryonic development. This finding aligns with a related study by Hao et al., who used whole-transcriptome sequencing in a POR mouse model and reported that acupuncture may regulate early embryonic development through the modulation of specific microRNAs and related signaling pathways (45).

This study enrolled patients meeting the Bologna criteria for POR, who presented with typical features of DOR, such as low AMH levels and a low AFC. In these patients, the depletion of the follicular pool and consequent lower inhibin B levels disrupt the negative feedback on the HPO axis, leading to a rise in basal FSH. Persistent high FSH downregulates FSH receptors on granulosa cells, causing FSH desensitization (46, 47). Thus, even high-dose FSH stimulation often fails, leading to uneven follicular growth, fewer oocytes, and fewer transferable embryos (48). Currently, for POR patients with elevated basal FSH levels, clinical practice commonly employs pretreatment with medications such as estrogens and progestogens, aiming to restore FSH receptor sensitivity by suppressing endogenous FSH, thereby improving synchronous follicular development. However, existing evidence indicates that while such regimens increase the dosage of exogenous medications and prolong the duration of ovarian stimulation, imposing a heavier economic burden on patients, their efficacy in improving follicular development and clinical pregnancy outcomes remains controversial (49, 50). In this context of limited options, this study provides evidence that acupuncture pretreatment can effectively lower basal FSH levels in women with POR, a finding consistent with several earlier RCTs (26, 51) and a systematic review (28).

### Strengths and limitations

This study has several key strengths. First, To our knowledge, the present study represents one of the largest multicenter randomized controlled trials conducted to date on acupuncture treatment for poor ovarian response. The robustness of the trial is ensured by a standardized acupuncture protocol with comprehensive training, a unified quality control system implemented throughout the study process, and a coordinated multicenter monitoring mechanism. Second, POR remains a major challenge in assisted reproduction, with a global prevalence of 5.6%-35.1% (17). This challenge is particularly acute in China, which performs an estimated 1.2 million IVF cycles annually (52). In this context, the observed, albeit non-significant, absolute improvements in total CPR (12.86%) and LBR (5.72%) are noteworthy. Given the large POR population and the paucity of effective therapies, these improvements could translate into a substantial number of additional pregnancies and live births at the national level. Finally, this study employed a standardized acupuncture protocol derived from long-term clinical practice. The acupoint selection primarily focused on the Ren meridian and Kidney meridian on the abdomen, and the Bladder meridian on the lumbosacral region, aiming to regulate the Chong and Ren meridians and tonify the liver and kidney. This was supplemented by acupoints on the lower limbs to regulate the liver, spleen, and kidney, and acupoints on the head to calm the mind. This protocol aligns with the core pathogenesis of DOR and POR, which is primarily seen as dysfunction of the Chong and Ren meridians and deficiency of the liver and kidney. The use of a fixed protocol minimizes inter-practitioner variability, which is particularly important for ensuring the consistency and reproducibility of the intervention in clinical studies.

This study has several limitations. First, the timeline was substantially prolonged due to repeated COVID-19-related disruptions in recruitment, treatment, and follow-up across all centers. Second, acupuncture did not yield significant improvements in key reproductive outcomes, such as retrieved oocyte count, clinical pregnancy, and live birth rates, which is possibly due to the limited sample size or treatment duration. Moreover, the absence of a sham-acupuncture control prevents the exclusion of potential placebo effects. Finally, the inclusion of only POR patients under the antagonist protocol may restrict the generalizability of our findings to other populations or COH regimens.

## Conclusions

In conclusion, this study did not demonstrate a significant improvement in the number of oocytes retrieved with acupuncture in patients with POR. Although acupuncture was associated with improved embryo cleavage rate and reduced basal FSH, these favorable laboratory findings did not translate into significant improvements in CPR or LBR. However, given the limited treatment options for POR and the potential increase in clinical pregnancy rate, further large-scale RCTs are warranted to verify the effect of acupuncture for POR.

## Data Availability

The raw data supporting the conclusions of this article will be made available by the authors, without undue reservation.
