# Supplementary material for: Effect of acupuncture for poor ovarian response: a multicenter randomized controlled trial
Source: Front Endocrinol (Lausanne). 2026 Feb 26;17:1765527. doi: 10.3389/fendo.2026.1765527 (PMC12979076; doi:10.3389/fendo.2026.1765527)
Supplement: Supplementary file 1 [file Table1.docx]

**Supplementary Table 1 The definition and calculation Methods of IVF Outcomes.**

|  | **Calculation Methods** |
| --- | --- |
| Fertilization rate | (Number of fertilized oocytes / Total number of oocytes retrieved) × 100% |
| Cleavage rate ^a^ | (Number of cleaved embryos / Number of fertilized oocytes) × 100% |
| Utilizable embryo rate | (Number of usable embryos / Number of cleaved embryos) × 100% |
| High-quality embryo rate | (Number of high-quality embryos / Number of cleaved embryos) × 100% |
| Embryo implantation rate | (Number of gestational sacs / Number of embryos transferred) × 100% |
| Total clinical pregnancy rate | (Number of clinical pregnancies achieved following fresh or frozen embryo transfer in the index ART cycle / Total number of women who received treatment) × 100% |
| Total live birth rate | (Number of live births achieved following fresh or frozen embryo transfer in the index ART cycle / Total number of women who received treatment) × 100% |
| Miscarriage rate | (Number of miscarriages / Number of clinical pregnancies) × 100 |

^a^Fertilization was assessed 16-18 hours later by examining the oocyte after removal of the cumulus and corona radiata cells. Normal fertilization was confirmed by the presence of two pronuclei (2PN) and two polar bodies, along with an intact zona pellucida and clear, evenly distributed cytoplasm.

**Supplementary Table 2 The outcomes in PP analysis.**

|  | **Acupuncture group**  **(*n* = 57)** | **Control**  **group**  **(*n* = 55)** | **Between-Group**  **Difference**  **(95% CI)** | ***P Value*** |
| --- | --- | --- | --- | --- |
| Number of oocytes retrieved(median (IQR)) | 2.00 (1.00-3.00) | 2.00 (1.00-4.00) | 0.00 (-1.00, 0.00) | 0.436 |
| Fertilization rate(No. (%)) | 88/114 (77.19) | 113/130 (86.92) | -9.73(-19.37, -0.09) | 0.005 |
| Embryo cleavage rate(No. (%)) | 87/88 (98.86) | 98/113 (86.73) | 12.14(5.5, 18.77) | 0.002 |
| Utilizable embryo rate(No. (%)) | 73/87 (83.91) | 81/98 (82.65) | 1.25(-9.51, 12.02) | 0.820 |
| Top-quality embryo rate(No. (%)) | 41/87 (47.13) | 43/81 (53.09) | -5.96(-21.06, 9.14) | 0.440 |
| Embryo implantation  rate(No. (%)) | 18/47 (38.30) | 16/45 (35.56) | 2.74(-16.97, 22.46) | 0.785 |
| Total CPR(No. (%)) | 19/57 (33.33) | 12/55 (21.82) | 11.52(-4.88, 27.91) | 0.173 |
| CPR per transfer fresh  (No. (%)) | 7/14 (50.00) | 7/12 (58.33) | -8.33 (-46.6, 29.93) | 0.713 |
| CPR per transfer FET  (No. (%)) | 12/18 (66.67) | 5/16 (31.25) | 35.42(3.95, 66.88) | 0.080 |
| Total LBR(No. (%)) | 10/57 (17.54) | 8/55 (14.55) | 3.00(-10.58, 16.57) | 0.666 |
| LBR per transfer fresh  (No. (%)) | 4/14 (28.57) | 6/12 (50.00) | -21.43(-58.31, 15.45) | 0.415 |
| LBR per transfer FET  (No. (%)) | 6/18 (33.33) | 2/16 (12.50) | 20.83(-6.31, 47.98) | 0.233 |
| Miscarriage rate  (No. (%)) | 9/19 (47.37) | 4/12 (33.33) | 14.04(-20.83, 48.9) | 0.441 |
| Regular menstruation  (No. (%)) | 39/57(68.4) | 33/55(60) | 8.40(-11.1, 27.9) | 0.431 |
| AFC(median (IQR)) | 4.00 (3.00-5.00) | 4.00 (3.00-6.00) | 0.00 (-1.00, 1.00) | 0.799 |
| AMH(ng/ml,median (IQR)) | 0.72 (0.38-1.09) | 0.76 (0.42-1.18) | -0.04 (-0.38, 0.25) | 0.757 |
| Basal FSH  (IU/L,median (IQR)) | 8.77 (6.4-12.74) | 10.83 (8.05-15.84) | -2.16 (-4.51, -0.29) | 0.035 |
| Basal LH  (IU/L,median (IQR)) | 3.29 (2.24-4.88) | 3.75 (2.9-5.16) | -0.55 (-1.21, 0.15) | 0.136 |
| Basal E_2_  (pg/ml,median (IQR)) | 59.00 (39.9-93.05) | 53.00 (37-79) | 7.08 (-6.20, 22.01) | 0.278 |
| SAS  (mean(SD)) | 31.6 (6.14) | 33.53 (7.74) | -1.94 (-4.64,0.76) | 0.158 |

SD, standard deviation; IQR, Interquartile Range; CPR, clinical pregnancy rate; LBR, live birth rate; AFC, antral follicle count; AMH, anti-Müllerian hormone; FSH, follicle-stimulating hormone; LH, luteinizing hormone; SAS, Self-Rating Anxiety Scale.

**Supplementary Table 3 Conditions of spontaneous conceptions.**

|  | **Number of natural pregnancy*** | **Number of live births** | **Mean gestational age at delivery for live births (weeks)** | **Mean number of acupuncture sessions per patient** |
| --- | --- | --- | --- | --- |
| Acupuncture group  (*n* = 70) | 3 | 3 | 38 | 33 |

*****Natural pregnancy was defined as a clinical pregnancy confirmed through natural conception, without the need for embryo transfer.
